# Home Life

**Published:** 1920-05-15

**Authors:** 


THE PUBLIC WELL-BEING—(*continued*)

### HOME LIFE.

### Striking Illustration in Vital Statistics.

A GOOD deal of attention has been devoted to health subjects during Health Week and Baby Week, which has just closed. Municipalities, the churches, and the Press have dealt with them from various angles, and a good many valuable common-sense hints have been given the public in such a form as to set them thinking. Of all that one has read one would place high in the list a lecture given at Bristol by Dr. C. W. Saleeby on the subject of home life in relation to the proper upbringing of children. He illustrated his contention that there is no substitute for homes by a striking contrast between Bradford and rural Ireland. (It is delightful to be able to go to Ireland for at least one favourable comparison.)

Bradford is a great industrial city, enlightened, well-informed, with a splendid municipality, with a greater expenditure than any other municipality in the world in proportion to the number of children cared for in the institutions looked after by splendid officers. The results in Bradford have been disappointing, despite the fact that improvements of every kind conceivable that would assist child life have been made. In recent years it has been impossible to find anything that a municipality could do that Bradford has not done. Everything available has been forthcoming—money, knowledge, devotion, and apparatus. Still the results have been miserable; so much so that even before the war the death-rate of the city was approaching very close to its extremely low birth-rate, whilst for the last two years the death-rate has been considerably higher than the birth-rate. The infant mortality was 135 per 1,000, with every modern advantage to combat it. At the same time the birth-rate of 13 per 1,000 was an extreme contrast in the civilisation or social form that appertained to the industrial centres and the rural districts of these islands. In Connaught there are ignorance, a minimum of medical resources, no institutions or anything that Bradford has provided for its infants. In Connaught there is an extremely high birth-rate, enormous families, far and away the highest in the country. In many parts of Connaught the birth-rate is between 45 and 50 per 1,000, and so high is the birth-rate that a large infant mortality would be expected amongst so many children. The mortality ranged round some such figure as 35 per 1,000.

Dr. Saleeby's deduction was that where there were no homes children perished, and that was the case in Bradford, whereas in Connaught, if there was nothing else, there was complete motherhood. There was no substitute for home, and by that he did not mean the sanitary building in which a family lived, but the home life created by a woman. In Connaught there was a rate of infant mortality that shamed the doctors of England. There was no compensating for the mother and her utility. This is all true, though Dr. Saleeby must also make allowance for other factors, such as air pollution, in any consideration of vital statistics in industrial cities.

Answering questions, Dr. Saleeby said the case for infant life in Bradford was made hopeless by the ruination of the home life, for there hundreds of married women went to work daily. He advocated most strongly that such a thing was wrong. Dr. Saleeby's view in this respect is admitted, to some extent, by conclusions reached at the international labour conference in Washington last year, and one of the results of this is that the British Government is shortly introducing a Bill to regulate, at any rate, the employment of women before and after child-birth. It does not meet the whole of Dr. Saleeby's point, but it goes a step towards it without raising the great and contentious problem of the right to work of married women.

